# 2-{2-[4-(4-Fluoro­phen­yl)piperazin-1-yl]-2-oxoeth­yl}-6-(morpholin-4-yl)-4-phenyl­pyridazin-3(2*H*)-one

**DOI:** 10.1107/S1600536811005071

**Published:** 2011-02-19

**Authors:** Abdullah Aydın, Murat Şüküroğlu, Mehmet Akkurt, Orhan Büyükgüngör

**Affiliations:** a Department of Science Education, Faculty of Education, Kastamonu University, 37200 Kastamonu, Turkey; b Department of Pharmaceutical Chemistry, Faculty of Pharmacy, Gazi University, 06330 Ankara, Turkey; c Department of Physics, Faculty of Sciences, Erciyes University, 38039 Kayseri, Turkey; d Department of Physics, Faculty of Arts and Sciences, Ondokuz Mayıs University, 55139 Samsun, Turkey

## Abstract

In the title compound, C_26_H_28_FN_5_O_3_, the morpholine ring adopts a chair conformation. The piperazine ring is puckered [*Q*_T_ = 0.5437 (15) Å, θ = 8.89 (15) and ϕ = 357.2 (11)°]. The 1,6-dihydro­pyridazine ring makes dihedral angles of 28.03 (7) and 77.46 (7)° with the phenyl and benzene rings, respectively. In the crystal, mol­ecules are linked along the *c* axis by C—H⋯O inter­actions and are flattened parallel to the *ac* plane. C–H⋯π inter­actions also contribute to the stability of the structure.

## Related literature

For the pharmacological effects, biological activity and synthesis of 3(2*H*)-pyridazinones, see: Şüküroğlu *et al.* 2006 ▶; Brogden 1986 ▶. For bond-length data, see: Allen *et al.* (1987 ▶). For ring conformational analysis, see: Cremer & Pople (1975 ▶). For the quantum mechanical *CNDO*/2 approximation, see: Pople & Beveridge (1970 ▶).
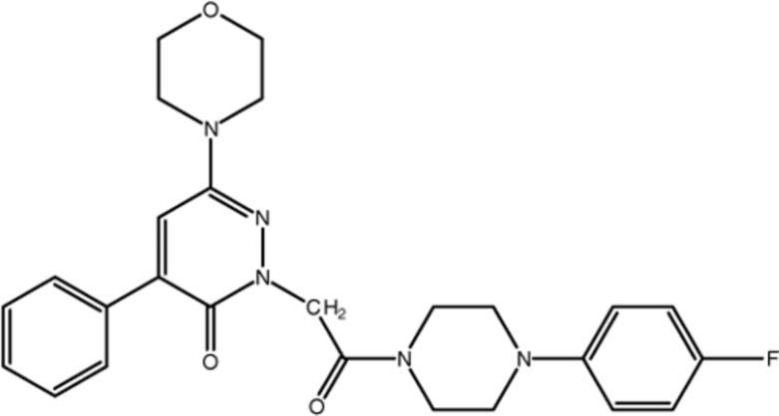


## Experimental

### 

#### Crystal data

C_26_H_28_FN_5_O_3_
*M* *_r_* = 477.53
Triclinic, 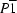

*a* = 8.9168 (5) Å
*b* = 10.7106 (6) Å
*c* = 13.5147 (8) Å
α = 73.489 (4)°
β = 71.309 (4)°
γ = 83.486 (4)°
*V* = 1171.87 (12) Å^3^
*Z* = 2
Mo *K*α radiation
μ = 0.10 mm^−1^
*T* = 296 K
0.60 × 0.49 × 0.20 mm

#### Data collection

STOE IPDS 2 diffractometer
Absorption correction: integration (*X-RED32*; Stoe & Cie, 2002 ▶) *T*_min_ = 0.945, *T*_max_ = 0.981
13273 measured reflections
4861 independent reflections
3479 reflections with *I* > 2σ(*I*)
*R*_int_ = 0.029

#### Refinement

*R*[*F* ^2^ > 2σ(*F* ^2^)] = 0.036
*wR*(*F* ^2^) = 0.091
*S* = 1.03
4861 reflections
316 parameters
H-atom parameters constrained
Δρ_max_ = 0.12 e Å^−3^
Δρ_min_ = −0.14 e Å^−3^

### 

Data collection: *X-AREA* (Stoe & Cie, 2002 ▶); cell refinement: *X-AREA*; data reduction: *X-RED32* (Stoe & Cie, 2002 ▶); program(s) used to solve structure: *SIR97* (Altomare *et al.*, 1999 ▶); program(s) used to refine structure: *SHELXL97* (Sheldrick, 2008 ▶); molecular graphics: *ORTEP-3 for Windows* (Farrugia, 1997 ▶); software used to prepare material for publication: *WinGX* (Farrugia, 1999 ▶).

## Supplementary Material

Crystal structure: contains datablocks global, I. DOI: 10.1107/S1600536811005071/ez2227sup1.cif

Structure factors: contains datablocks I. DOI: 10.1107/S1600536811005071/ez2227Isup2.hkl

Additional supplementary materials:  crystallographic information; 3D view; checkCIF report

## Figures and Tables

**Table 1:** Hydrogen-bond geometry (Å, °) *Cg*2, *Cg*4 and *Cg*5 are the centroids of the N1/N2/C7–C10, C1–C6 and C21–C26 rings, respectively.

*D*—H⋯*A*	*D*—H	H⋯*A*	*D*⋯*A*	*D*—H⋯*A*
C11—H11*B*⋯O3^i^	0.97	2.41	3.3306 (18)	159
C5—H5⋯*Cg*5^ii^	0.93	2.86	3.4941 (18)	127
C13—H13*B*⋯*Cg*4^i^	0.97	2.92	3.7395 (19)	143
C18—H18*A*⋯*Cg*2^iii^	0.97	2.73	3.5079 (16)	138

Symmetry codes: (i) 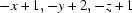; (ii) 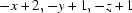; (iii) 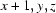.

**Fig. 1.**
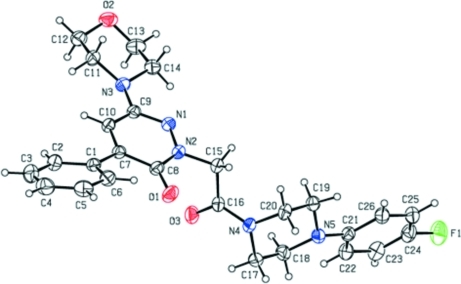
View of the title molecule with the atom numbering scheme. Displacement ellipsoids for non-H atoms are drawn at the 30% probability level.

**Fig. 2.**
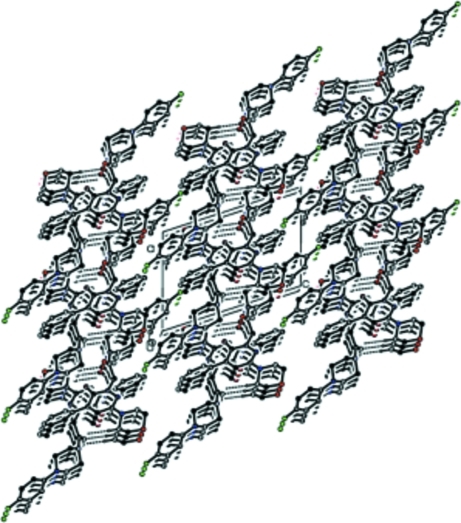
The packing and hydrogen bonding interactions of (I) down the b axis. H atoms not participating in hydrogen bonding have been omitted for clarity.

**Fig. 3.**
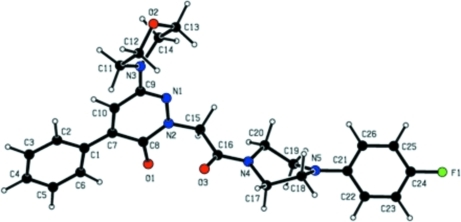
The spatial view of the title molecule (I), calculated by the CNDO/2 aproximation.

## supplementary crystallographic information

### Comment

In recent years, the 3(2*H*)-pyridazinone system has aroused a great deal of attention due to its structural relationship to pyrazolone derivatives such as aminopyrine and dipyrone in view of the ring enlargement of pyrazolone to pyridazinone. These drugs possess analgesic and anti-inflammatory activities although they have limitations for their clinical use due to serious side effects such as blood dyscrasias (Şüküroğlu *et al.*, 2006; Brogden, 1986).

A series of 6-morpholino-4-aryl-3(2*H*)-pyridazinone alkanoic acids, their ester and amide derivatives were prepared and tested for their *in vivo* analgesic activity by using the *p*-benzoquinone-induced writhing test. The title compound, C_26_H_28_FN_5_O_3_, generally showed higher activity but caused gastric ulceration in the animals (Şüküroğlu *et al.*, 2006).

In the title molecule (I), Fig. 1, the morpholine ring (N3/O2/C11–C14) adopts a chair conformation. The piperazine ring (N4/N5/C17–C20) is puckered. The conformation of this ring is described by three puckering parameters: Q_T_ = 0.5437 (15) Å, θ = 8.89 (15) ° and, φ = 357.2 (11) ° (Cremer & Pople, 1975). The 1,6-dihydropyridazine ring (N1/N2/C7–C10) makes dihedral angles of 28.03 (7) and 77.46 (7) ° with the C1–C6 phenyl and C21–C26 benzene rings, respectively. The phenyl and benzene rings make a dihedral angle of 50.17 (8) ° with each other. In the crystal structure, molecules are linked along the *c*-axis direction and are flattened parallel to the plane containing the a and c axes. Furthermore, C–H···π interactions contribute to the stability of the structure (Table 1). Fig. 2 shows the packing diagram of (I) down the *b* axis.

Theoretical calculations were carried out using the semiempirical quantum-mechanical *CNDO*/2 (Complete Neglect of Differential Overlap) method (Pople and Beveridge, 1970). The spatial view of the single molecule calculated as closed-shell in a vacuum is shown in Fig. 3 with atomic labels. The calculated dipole moment of (I) is about 2.795 Debye. The *HOMO* and *LUMO* energy levels are -10.013 and 0.832 eV, respectively.

According to the theoretical *CNDO/2* and experimental X-ray structural results, the values of the geometric parameters of (I) are almost comparable within the experimental error interval (Allen *et al.*, 1987).

The 1,6-dihydropyridazine ring (N1/N2/C7–C10) forms dihedral angles of 2.24 and 60.48° with the C1–C6 phenyl and C21–C26 benzene rings, respectively. The dihedral angle between the phenyl and benzene rings is 62.62°. The orientations of the planes of the rings are however, slightly different in the *CNDO*/2 and X-ray results. That is, intermolecular interactions play an important role in determining the crystal state conformation of (I).

### Experimental

A reaction mixture containing 2-[4-phenyl-6-(morpholin-4-yl)-3(2*H*)- pyridazinone-2-yl]acetic acid (0.01 mole) and triethylamine (0.011 mole) in 20 ml dichloromethane at 273 K (ice-bath) was treated with ethyl chloroformate (0,01 mole). After stirring the reaction mixture at 273 K for 15 min, 0.011 mole of 4-(4-fluorophenyl)-piperazine derivative was added to this solution. The final mixture was stirred at room temperature for 24 h and evaporated to dryness and then acetone was added. All undissolved salts were filtered off, the filtrate was evaporated to dryness and the residue was recrystallized from acetone-water (1:1) to yield 62%, [m.p.: 457 K].

^1^H-NMR (CDCl_3_), δ 7.75 (m, 2H, phenyl-H2, H6), 7.43 (m, 3H, phenyl-H3, H4, H5), 7.23 (s, 1H, pyridazinone-H5), 6.99 (m, 4H, 4-fluorophenyl-H2, H3, H5, H6), 4.98 (s, 2H, N—CH_2_—CO), 3.82 (m, 6H, morpholine-H2, H6, piperazine- H2(6)), 3.71 (m, 2H, piperazine-H6(2)), 3.31 (t, 8H, morpholine-H3, H5), 3.15 (m, 4H, piperazine-H3, H5) p.p.m.. IR *v*_max_ cm^-1^ (KBr): 2845, 1661, 1643. Anal. C, H, N (C_26_H_28_FN_5_O_3_) (Şüküroğlu *et al.*, 2006). Elemental analysis: C_26_H_28_FN_5_O_3_, Calc.(%) / Found (%): C: 65.39/65.54, H: 5.91/5.49, N: 14.67/14.28.

### Refinement

All H atoms were positioned geometrically and refined using a riding model with C—H = 0.93 and 0.97 Å, and *U*_iso_(H) = 1.2*U*_eq_(C).

### Crystal data

**Table:**

C_26_H_28_FN_5_O_3_	*Z* = 2
*M**_r_* = 477.53	*F*(000) = 504
Triclinic, *P*1	*D*_x_ = 1.353 Mg m^−^^3^
Hall symbol: -P 1	Mo *K*α radiation, λ = 0.71073 Å
*a* = 8.9168 (5) Å	Cell parameters from 19046 reflections
*b* = 10.7106 (6) Å	θ = 1.7–28.2°
*c* = 13.5147 (8) Å	µ = 0.10 mm^−^^1^
α = 73.489 (4)°	*T* = 296 K
β = 71.309 (4)°	Prism, yellow
γ = 83.486 (4)°	0.60 × 0.49 × 0.20 mm
*V* = 1171.87 (12) Å^3^	

### Data collection

**Table:**

STOE IPDS 2 diffractometer	4861 independent reflections
Radiation source: sealed X-ray tube, 12 x 0.4 mm long-fine focus	3479 reflections with *I* > 2σ(*I*)
plane graphite	*R*_int_ = 0.029
Detector resolution: 6.67 pixels mm^-1^	θ_max_ = 26.5°, θ_min_ = 1.7°
ω scans	*h* = −11→11
Absorption correction: integration (*X-RED32*; Stoe & Cie, 2002)	*k* = −13→13
*T*_min_ = 0.945, *T*_max_ = 0.981	*l* = −16→16
13273 measured reflections	

### Refinement

**Table:**

Refinement on *F*^2^	Primary atom site location: structure-invariant direct methods
Least-squares matrix: full	Secondary atom site location: difference Fourier map
*R*[*F*^2^ > 2σ(*F*^2^)] = 0.036	Hydrogen site location: inferred from neighbouring sites
*wR*(*F*^2^) = 0.091	H-atom parameters constrained
*S* = 1.03	*w* = 1/[σ^2^(*F*_o_^2^) + (0.0469*P*)^2^ + 0.0141*P*] where *P* = (*F*_o_^2^ + 2*F*_c_^2^)/3
4861 reflections	(Δ/σ)_max_ < 0.001
316 parameters	Δρ_max_ = 0.12 e Å^−^^3^
0 restraints	Δρ_min_ = −0.14 e Å^−^^3^

### Special details

**Table:**

Geometry. Bond distances, angles *etc*. have been calculated using the rounded fractional coordinates. All su's are estimated from the variances of the (full) variance-covariance matrix. The cell e.s.d.'s are taken into account in the estimation of distances, angles and torsion angles
Refinement. Refinement on *F*^2^ for ALL reflections except those flagged by the user for potential systematic errors. Weighted *R*-factors *wR* and all goodnesses of fit *S* are based on *F*^2^, conventional *R*-factors *R* are based on *F*, with *F* set to zero for negative *F*^2^. The observed criterion of *F*^2^ > σ(*F*^2^) is used only for calculating -*R*-factor-obs *etc*. and is not relevant to the choice of reflections for refinement. *R*-factors based on *F*^2^ are statistically about twice as large as those based on *F*, and *R*-factors based on ALL data will be even larger.

### Fractional atomic coordinates and isotropic or equivalent isotropic displacement parameters (Å^2^)

**Table:**

	*x*	*y*	*z*	*U*_iso_*/*U*_eq_	
F1	1.31808 (14)	0.32221 (10)	1.12298 (8)	0.0875 (4)	
O1	0.65668 (12)	0.66791 (10)	0.43998 (8)	0.0635 (4)	
O2	0.02122 (12)	1.05391 (10)	0.84902 (8)	0.0626 (3)	
O3	0.84333 (12)	0.78850 (9)	0.56875 (9)	0.0627 (3)	
N1	0.44450 (12)	0.78805 (9)	0.66593 (8)	0.0437 (3)	
N2	0.54428 (12)	0.72130 (10)	0.59688 (8)	0.0438 (3)	
N3	0.25440 (13)	0.94880 (10)	0.69277 (8)	0.0471 (3)	
N4	0.93182 (13)	0.58003 (10)	0.61148 (9)	0.0473 (4)	
N5	1.09333 (13)	0.46726 (10)	0.76728 (8)	0.0459 (3)	
C1	0.43486 (15)	0.84428 (12)	0.33963 (10)	0.0425 (4)	
C2	0.38220 (17)	0.96206 (13)	0.28338 (11)	0.0530 (5)	
C3	0.3712 (2)	0.97650 (17)	0.18124 (13)	0.0668 (6)	
C4	0.4089 (2)	0.87442 (18)	0.13408 (12)	0.0671 (6)	
C5	0.46053 (19)	0.75785 (16)	0.18863 (12)	0.0622 (6)	
C6	0.47474 (17)	0.74228 (13)	0.29017 (11)	0.0523 (5)	
C7	0.44320 (14)	0.82808 (11)	0.45042 (10)	0.0402 (4)	
C8	0.55601 (15)	0.73397 (12)	0.49132 (10)	0.0445 (4)	
C9	0.35078 (14)	0.87626 (11)	0.62510 (10)	0.0406 (4)	
C10	0.34782 (15)	0.89739 (11)	0.51700 (10)	0.0429 (4)	
C11	0.10441 (17)	1.00413 (13)	0.67498 (11)	0.0546 (5)	
C12	0.04171 (18)	1.10537 (13)	0.73676 (12)	0.0615 (5)	
C13	0.17058 (19)	1.00733 (16)	0.86405 (13)	0.0641 (6)	
C14	0.24172 (18)	0.90257 (14)	0.80774 (11)	0.0533 (5)	
C15	0.64791 (15)	0.62509 (12)	0.64412 (11)	0.0462 (4)	
C16	0.81643 (16)	0.67228 (12)	0.60443 (10)	0.0444 (4)	
C17	1.09342 (16)	0.61613 (15)	0.59093 (11)	0.0578 (5)	
C18	1.12789 (17)	0.59830 (13)	0.69637 (12)	0.0553 (5)	
C19	0.93822 (16)	0.42227 (12)	0.78027 (11)	0.0460 (4)	
C20	0.90918 (17)	0.44392 (12)	0.67254 (11)	0.0484 (4)	
C21	1.14574 (15)	0.43318 (12)	0.85944 (10)	0.0446 (4)	
C22	1.24267 (18)	0.51424 (13)	0.87566 (12)	0.0532 (5)	
C23	1.2999 (2)	0.47658 (15)	0.96358 (13)	0.0611 (6)	
C24	1.26051 (19)	0.35931 (15)	1.03610 (12)	0.0599 (5)	
C25	1.16453 (19)	0.27695 (15)	1.02480 (12)	0.0596 (5)	
C26	1.10713 (17)	0.31386 (13)	0.93713 (11)	0.0530 (5)	
H2	0.35420	1.03140	0.31490	0.0640*	
H3	0.33800	1.05620	0.14380	0.0800*	
H4	0.39940	0.88440	0.06570	0.0810*	
H5	0.48620	0.68860	0.15690	0.0750*	
H6	0.51130	0.66300	0.32590	0.0630*	
H10	0.27860	0.96040	0.49140	0.0510*	
H11A	0.02810	0.93570	0.69910	0.0660*	
H11B	0.12070	1.04350	0.59830	0.0660*	
H12A	0.11470	1.17670	0.70830	0.0740*	
H12B	−0.05910	1.13980	0.72630	0.0740*	
H13A	0.15730	0.97320	0.94090	0.0770*	
H13B	0.24260	1.07930	0.83630	0.0770*	
H14A	0.34600	0.87750	0.81590	0.0640*	
H14B	0.17600	0.82650	0.84070	0.0640*	
H15A	0.64680	0.54450	0.62510	0.0550*	
H15B	0.60890	0.60740	0.72240	0.0550*	
H17A	1.10820	0.70640	0.54920	0.0690*	
H17B	1.16660	0.56240	0.54900	0.0690*	
H18A	1.23860	0.61530	0.68140	0.0660*	
H18B	1.06480	0.66110	0.73320	0.0660*	
H19A	0.85690	0.46850	0.82480	0.0550*	
H19B	0.93100	0.33020	0.81730	0.0550*	
H20A	0.98170	0.38910	0.63140	0.0580*	
H20B	0.80200	0.41990	0.68420	0.0580*	
H22	1.26910	0.59500	0.82640	0.0640*	
H23	1.36500	0.53130	0.97310	0.0730*	
H25	1.13830	0.19710	1.07550	0.0720*	
H26	1.04120	0.25820	0.92940	0.0630*	

### Atomic displacement parameters (Å^2^)

**Table:**

	*U*^11^	*U*^22^	*U*^33^	*U*^12^	*U*^13^	*U*^23^
F1	0.1156 (9)	0.0924 (7)	0.0722 (6)	0.0160 (6)	−0.0589 (6)	−0.0215 (5)
O1	0.0598 (7)	0.0678 (6)	0.0611 (6)	0.0271 (5)	−0.0168 (5)	−0.0268 (5)
O2	0.0534 (6)	0.0630 (6)	0.0617 (6)	0.0020 (5)	0.0024 (5)	−0.0255 (5)
O3	0.0606 (6)	0.0408 (5)	0.0794 (7)	−0.0021 (4)	−0.0271 (5)	0.0026 (5)
N1	0.0414 (6)	0.0417 (5)	0.0441 (6)	0.0032 (4)	−0.0094 (5)	−0.0111 (5)
N2	0.0391 (6)	0.0427 (5)	0.0463 (6)	0.0082 (4)	−0.0124 (5)	−0.0108 (5)
N3	0.0453 (6)	0.0442 (6)	0.0463 (6)	0.0071 (5)	−0.0075 (5)	−0.0140 (5)
N4	0.0395 (6)	0.0438 (6)	0.0548 (7)	0.0038 (5)	−0.0182 (5)	−0.0045 (5)
N5	0.0445 (6)	0.0450 (6)	0.0459 (6)	−0.0017 (5)	−0.0163 (5)	−0.0053 (5)
C1	0.0356 (7)	0.0440 (7)	0.0459 (7)	−0.0017 (5)	−0.0097 (5)	−0.0114 (5)
C2	0.0543 (9)	0.0500 (8)	0.0552 (8)	0.0054 (6)	−0.0206 (7)	−0.0127 (6)
C3	0.0701 (11)	0.0693 (10)	0.0592 (9)	0.0068 (8)	−0.0283 (8)	−0.0077 (8)
C4	0.0657 (11)	0.0914 (12)	0.0465 (8)	−0.0052 (9)	−0.0193 (7)	−0.0179 (8)
C5	0.0626 (10)	0.0713 (10)	0.0561 (9)	−0.0032 (8)	−0.0116 (7)	−0.0288 (8)
C6	0.0543 (9)	0.0508 (8)	0.0516 (8)	0.0003 (6)	−0.0137 (6)	−0.0166 (6)
C7	0.0357 (7)	0.0375 (6)	0.0452 (7)	−0.0016 (5)	−0.0101 (5)	−0.0095 (5)
C8	0.0389 (7)	0.0434 (7)	0.0497 (7)	0.0048 (5)	−0.0112 (6)	−0.0145 (6)
C9	0.0371 (7)	0.0360 (6)	0.0450 (7)	−0.0004 (5)	−0.0082 (5)	−0.0100 (5)
C10	0.0388 (7)	0.0386 (6)	0.0492 (7)	0.0048 (5)	−0.0136 (5)	−0.0102 (5)
C11	0.0529 (9)	0.0497 (7)	0.0521 (8)	0.0148 (6)	−0.0100 (6)	−0.0122 (6)
C12	0.0565 (9)	0.0448 (7)	0.0671 (10)	0.0079 (6)	−0.0004 (7)	−0.0142 (7)
C13	0.0578 (10)	0.0736 (10)	0.0607 (9)	−0.0014 (8)	−0.0060 (7)	−0.0310 (8)
C14	0.0518 (8)	0.0563 (8)	0.0478 (8)	0.0018 (6)	−0.0088 (6)	−0.0158 (6)
C15	0.0420 (7)	0.0415 (6)	0.0503 (7)	0.0058 (5)	−0.0156 (6)	−0.0057 (6)
C16	0.0471 (8)	0.0411 (7)	0.0432 (7)	0.0035 (5)	−0.0178 (6)	−0.0053 (5)
C17	0.0400 (8)	0.0661 (9)	0.0545 (8)	−0.0036 (6)	−0.0137 (6)	0.0039 (7)
C18	0.0478 (8)	0.0533 (8)	0.0591 (9)	−0.0096 (6)	−0.0200 (7)	0.0015 (7)
C19	0.0476 (8)	0.0372 (6)	0.0512 (7)	0.0004 (5)	−0.0142 (6)	−0.0100 (5)
C20	0.0513 (8)	0.0384 (6)	0.0588 (8)	0.0083 (5)	−0.0240 (6)	−0.0131 (6)
C21	0.0442 (7)	0.0434 (7)	0.0448 (7)	0.0072 (5)	−0.0134 (6)	−0.0126 (5)
C22	0.0596 (9)	0.0455 (7)	0.0574 (8)	0.0032 (6)	−0.0224 (7)	−0.0144 (6)
C23	0.0685 (10)	0.0594 (9)	0.0690 (10)	0.0083 (7)	−0.0328 (8)	−0.0282 (8)
C24	0.0682 (10)	0.0666 (9)	0.0525 (8)	0.0179 (8)	−0.0302 (7)	−0.0213 (7)
C25	0.0634 (10)	0.0571 (8)	0.0518 (8)	0.0062 (7)	−0.0197 (7)	−0.0045 (7)
C26	0.0534 (9)	0.0490 (7)	0.0551 (8)	0.0000 (6)	−0.0203 (7)	−0.0077 (6)

### Geometric parameters (Å, °)

**Table:**

F1—C24	1.369 (2)	C21—C26	1.3999 (19)
O1—C8	1.2343 (17)	C22—C23	1.380 (2)
O2—C12	1.4173 (18)	C23—C24	1.357 (2)
O2—C13	1.424 (2)	C24—C25	1.366 (2)
O3—C16	1.2215 (17)	C25—C26	1.378 (2)
N1—N2	1.3698 (15)	C2—H2	0.9300
N1—C9	1.3098 (17)	C3—H3	0.9300
N2—C8	1.3646 (16)	C4—H4	0.9300
N2—C15	1.4531 (18)	C5—H5	0.9300
N3—C9	1.3883 (16)	C6—H6	0.9300
N3—C11	1.464 (2)	C10—H10	0.9300
N3—C14	1.4612 (17)	C11—H11A	0.9700
N4—C16	1.3501 (18)	C11—H11B	0.9700
N4—C17	1.4532 (19)	C12—H12A	0.9700
N4—C20	1.4571 (17)	C12—H12B	0.9700
N5—C18	1.4586 (18)	C13—H13A	0.9700
N5—C19	1.4572 (19)	C13—H13B	0.9700
N5—C21	1.4067 (17)	C14—H14A	0.9700
C1—C2	1.393 (2)	C14—H14B	0.9700
C1—C6	1.3933 (19)	C15—H15A	0.9700
C1—C7	1.4827 (18)	C15—H15B	0.9700
C2—C3	1.379 (2)	C17—H17A	0.9700
C3—C4	1.375 (3)	C17—H17B	0.9700
C4—C5	1.371 (3)	C18—H18A	0.9700
C5—C6	1.380 (2)	C18—H18B	0.9700
C7—C8	1.4639 (19)	C19—H19A	0.9700
C7—C10	1.3528 (18)	C19—H19B	0.9700
C9—C10	1.4219 (18)	C20—H20A	0.9700
C11—C12	1.504 (2)	C20—H20B	0.9700
C13—C14	1.502 (2)	C22—H22	0.9300
C15—C16	1.518 (2)	C23—H23	0.9300
C17—C18	1.508 (2)	C25—H25	0.9300
C19—C20	1.509 (2)	C26—H26	0.9300
C21—C22	1.396 (2)		
			
F1···H3^i^	2.7700	H2···H10	2.1900
F1···H15B^ii^	2.6900	H2···O3^iii^	2.9100
F1···H19A^ii^	2.7100	H2···N1^iii^	2.9000
O1···C6	2.8742 (19)	H3···F1^xii^	2.7700
O1···C16	3.0123 (18)	H3···C25^xii^	3.0300
O2···N3	2.8383 (15)	H3···H25^xii^	2.4400
O3···N2	2.7209 (16)	H5···C22^iv^	3.0700
O3···C11^iii^	3.3306 (18)	H5···C23^iv^	2.8800
O3···C8	3.2257 (18)	H5···C24^iv^	2.9800
O1···H12A^iii^	2.6800	H6···O1	2.3200
O1···H15A	2.4500	H6···C8	2.7100
O1···H6	2.3200	H6···H15A^xi^	2.5800
O1···H17B^iv^	2.7700	H10···C2	2.6600
O2···H13A^v^	2.7200	H10···C11	2.6100
O2···H26^vi^	2.7500	H10···H2	2.1900
O3···H17A	2.3800	H10···H11B	2.0000
O3···H2^iii^	2.9100	H10···O3^iii^	2.7800
O3···H10^iii^	2.7800	H11B···C10	2.5700
O3···H11B^iii^	2.4100	H11B···H10	2.0000
N2···O3	2.7209 (16)	H11B···O3^iii^	2.4100
N3···O2	2.8383 (15)	H12A···H13B	2.3100
N4···N5	2.8407 (16)	H12A···H20A^vi^	2.5500
N5···N4	2.8407 (16)	H12A···O1^iii^	2.6800
N1···H22^vii^	2.7300	H12B···C3^x^	2.9200
N1···H2^iii^	2.9000	H12B···C4^x^	3.0800
N1···H18A^vii^	2.6700	H13A···O2^v^	2.7200
N1···H14A	2.3600	H13B···H12A	2.3100
N1···H14B	2.8600	H13B···C6^iii^	3.0700
N2···H18A^vii^	2.8300	H14A···N1	2.3600
C2···C14^iii^	3.508 (2)	H14A···C2^iii^	2.8600
C6···O1	2.8742 (19)	H14B···N1	2.8600
C7···C9^iii^	3.5673 (18)	H14B···H22^vii^	2.5600
C8···O3	3.2257 (18)	H15A···O1	2.4500
C9···C7^iii^	3.5673 (18)	H15A···C20	2.6500
C9···C18^vii^	3.497 (2)	H15A···H20B	2.0000
C9···C10^iii^	3.5089 (18)	H15A···H6^xi^	2.5800
C10···C9^iii^	3.5089 (18)	H15B···C20	3.0200
C10···C10^iii^	3.5197 (19)	H15B···H20B	2.5500
C11···O3^iii^	3.3306 (18)	H15B···F1^ii^	2.6900
C12···C19^vi^	3.577 (2)	H17A···O3	2.3800
C14···C2^iii^	3.508 (2)	H17A···C10^viii^	2.9700
C16···O1	3.0123 (18)	H17B···H20A	2.4000
C18···C9^viii^	3.497 (2)	H17B···O1^iv^	2.7700
C19···C12^ix^	3.577 (2)	H18A···N1^viii^	2.6700
C2···H14A^iii^	2.8600	H18A···N2^viii^	2.8300
C2···H10	2.6600	H18A···C9^viii^	2.8900
C3···H12B^x^	2.9200	H18A···C22	2.5500
C4···H12B^x^	3.0800	H18A···H22	2.0100
C5···H20B^xi^	2.9400	H18B···C22	2.8900
C6···H20B^xi^	3.0500	H18B···H22	2.4700
C6···H13B^iii^	3.0700	H19A···F1^ii^	2.7100
C8···H6	2.7100	H19A···C23^ii^	2.9500
C9···H18A^vii^	2.8900	H19A···C24^ii^	2.8800
C10···H17A^vii^	2.9700	H19B···C12^ix^	2.8700
C10···H2	2.6900	H19B···C26	2.5500
C10···H11B	2.5700	H19B···H26	1.9900
C11···H10	2.6100	H20A···C12^ix^	3.0300
C12···H20A^vi^	3.0300	H20A···H12A^ix^	2.5500
C12···H19B^vi^	2.8700	H20A···H17B	2.4000
C13···H26^vi^	3.0500	H20B···C15	2.4800
C15···H20B	2.4800	H20B···H15A	2.0000
C18···H22	2.4600	H20B···H15B	2.5500
C19···H26	2.6100	H20B···C5^xi^	2.9400
C20···H15A	2.6500	H20B···C6^xi^	3.0500
C20···H15B	3.0200	H22···N1^viii^	2.7300
C22···H18A	2.5500	H22···C18	2.4600
C22···H18B	2.8900	H22···H14B^viii^	2.5600
C22···H5^iv^	3.0700	H22···H18A	2.0100
C23···H5^iv^	2.8800	H22···H18B	2.4700
C23···H19A^ii^	2.9500	H25···H3^i^	2.4400
C24···H5^iv^	2.9800	H26···O2^ix^	2.7500
C24···H19A^ii^	2.8800	H26···C13^ix^	3.0500
C25···H3^i^	3.0300	H26···C19	2.6100
C26···H19B	2.5500	H26···H19B	1.9900
H2···C10	2.6900		
			
C12—O2—C13	108.95 (12)	C4—C5—H5	120.00
N2—N1—C9	116.01 (10)	C6—C5—H5	120.00
N1—N2—C8	127.91 (11)	C1—C6—H6	120.00
N1—N2—C15	114.75 (10)	C5—C6—H6	120.00
C8—N2—C15	117.34 (11)	C7—C10—H10	119.00
C9—N3—C11	119.14 (11)	C9—C10—H10	119.00
C9—N3—C14	117.13 (11)	N3—C11—H11A	110.00
C11—N3—C14	112.25 (11)	N3—C11—H11B	110.00
C16—N4—C17	120.60 (12)	C12—C11—H11A	110.00
C16—N4—C20	126.08 (12)	C12—C11—H11B	110.00
C17—N4—C20	110.06 (12)	H11A—C11—H11B	108.00
C18—N5—C19	114.00 (11)	O2—C12—H12A	109.00
C18—N5—C21	116.78 (11)	O2—C12—H12B	109.00
C19—N5—C21	117.13 (10)	C11—C12—H12A	109.00
C2—C1—C6	118.22 (12)	C11—C12—H12B	109.00
C2—C1—C7	120.29 (12)	H12A—C12—H12B	108.00
C6—C1—C7	121.46 (12)	O2—C13—H13A	109.00
C1—C2—C3	120.40 (14)	O2—C13—H13B	109.00
C2—C3—C4	120.71 (16)	C14—C13—H13A	109.00
C3—C4—C5	119.50 (15)	C14—C13—H13B	109.00
C4—C5—C6	120.59 (15)	H13A—C13—H13B	108.00
C1—C6—C5	120.57 (14)	N3—C14—H14A	110.00
C1—C7—C8	120.11 (11)	N3—C14—H14B	110.00
C1—C7—C10	122.01 (12)	C13—C14—H14A	110.00
C8—C7—C10	117.88 (11)	C13—C14—H14B	110.00
O1—C8—N2	119.12 (12)	H14A—C14—H14B	108.00
O1—C8—C7	126.40 (12)	N2—C15—H15A	109.00
N2—C8—C7	114.49 (11)	N2—C15—H15B	109.00
N1—C9—N3	116.54 (11)	C16—C15—H15A	109.00
N1—C9—C10	121.96 (11)	C16—C15—H15B	109.00
N3—C9—C10	121.49 (11)	H15A—C15—H15B	108.00
C7—C10—C9	121.60 (12)	N4—C17—H17A	110.00
N3—C11—C12	109.61 (12)	N4—C17—H17B	110.00
O2—C12—C11	112.03 (12)	C18—C17—H17A	110.00
O2—C13—C14	112.09 (14)	C18—C17—H17B	110.00
N3—C14—C13	110.40 (12)	H17A—C17—H17B	108.00
N2—C15—C16	111.27 (11)	N5—C18—H18A	109.00
O3—C16—N4	122.80 (14)	N5—C18—H18B	109.00
O3—C16—C15	120.54 (13)	C17—C18—H18A	109.00
N4—C16—C15	116.67 (11)	C17—C18—H18B	109.00
N4—C17—C18	110.23 (12)	H18A—C18—H18B	108.00
N5—C18—C17	112.17 (12)	N5—C19—H19A	109.00
N5—C19—C20	111.62 (11)	N5—C19—H19B	109.00
N4—C20—C19	110.40 (11)	C20—C19—H19A	109.00
N5—C21—C22	121.73 (12)	C20—C19—H19B	109.00
N5—C21—C26	121.14 (12)	H19A—C19—H19B	108.00
C22—C21—C26	117.10 (13)	N4—C20—H20A	110.00
C21—C22—C23	121.10 (14)	N4—C20—H20B	110.00
C22—C23—C24	119.61 (16)	C19—C20—H20A	110.00
F1—C24—C23	119.38 (15)	C19—C20—H20B	110.00
F1—C24—C25	118.95 (14)	H20A—C20—H20B	108.00
C23—C24—C25	121.67 (15)	C21—C22—H22	119.00
C24—C25—C26	119.02 (14)	C23—C22—H22	119.00
C21—C26—C25	121.49 (14)	C22—C23—H23	120.00
C1—C2—H2	120.00	C24—C23—H23	120.00
C3—C2—H2	120.00	C24—C25—H25	121.00
C2—C3—H3	120.00	C26—C25—H25	120.00
C4—C3—H3	120.00	C21—C26—H26	119.00
C3—C4—H4	120.00	C25—C26—H26	119.00
C5—C4—H4	120.00		
			
C13—O2—C12—C11	60.78 (16)	C6—C1—C7—C10	−151.12 (14)
C12—O2—C13—C14	−59.74 (16)	C6—C1—C7—C8	28.3 (2)
C9—N1—N2—C8	−1.23 (19)	C6—C1—C2—C3	−0.4 (2)
N2—N1—C9—C10	2.71 (18)	C7—C1—C2—C3	−178.64 (14)
C9—N1—N2—C15	179.39 (11)	C2—C1—C7—C8	−153.52 (13)
N2—N1—C9—N3	−177.06 (11)	C2—C1—C6—C5	−0.7 (2)
C15—N2—C8—O1	−2.74 (19)	C7—C1—C6—C5	177.56 (14)
N1—N2—C8—C7	−2.13 (19)	C2—C1—C7—C10	27.1 (2)
C15—N2—C8—C7	177.24 (11)	C1—C2—C3—C4	1.3 (3)
N1—N2—C15—C16	−105.23 (12)	C2—C3—C4—C5	−1.1 (3)
C8—N2—C15—C16	75.32 (14)	C3—C4—C5—C6	0.1 (3)
N1—N2—C8—O1	177.89 (12)	C4—C5—C6—C1	0.9 (3)
C14—N3—C11—C12	52.25 (15)	C1—C7—C10—C9	176.59 (12)
C14—N3—C9—N1	−12.56 (18)	C8—C7—C10—C9	−2.82 (19)
C14—N3—C9—C10	167.66 (13)	C1—C7—C8—O1	4.6 (2)
C9—N3—C14—C13	165.09 (13)	C10—C7—C8—N2	3.99 (18)
C11—N3—C14—C13	−51.70 (16)	C1—C7—C8—N2	−175.43 (12)
C9—N3—C11—C12	−165.36 (11)	C10—C7—C8—O1	−176.03 (14)
C11—N3—C9—N1	−153.17 (12)	N1—C9—C10—C7	−0.7 (2)
C11—N3—C9—C10	27.06 (18)	N3—C9—C10—C7	179.07 (12)
C16—N4—C20—C19	−98.68 (16)	N3—C11—C12—O2	−57.26 (16)
C17—N4—C16—O3	8.9 (2)	O2—C13—C14—N3	55.52 (17)
C17—N4—C16—C15	−170.73 (11)	N2—C15—C16—N4	−158.32 (11)
C20—N4—C16—C15	−13.24 (19)	N2—C15—C16—O3	22.07 (17)
C20—N4—C17—C18	−60.28 (15)	N4—C17—C18—N5	53.86 (16)
C16—N4—C17—C18	100.50 (15)	N5—C19—C20—N4	−54.43 (15)
C20—N4—C16—O3	166.37 (13)	N5—C21—C22—C23	−176.61 (14)
C17—N4—C20—C19	60.79 (15)	C26—C21—C22—C23	1.2 (2)
C19—N5—C18—C17	−48.75 (16)	N5—C21—C26—C25	176.70 (14)
C21—N5—C19—C20	−169.67 (11)	C22—C21—C26—C25	−1.1 (2)
C19—N5—C21—C26	34.26 (18)	C21—C22—C23—C24	−0.5 (2)
C18—N5—C19—C20	48.87 (15)	C22—C23—C24—F1	179.76 (15)
C18—N5—C21—C26	174.65 (13)	C22—C23—C24—C25	−0.3 (3)
C18—N5—C21—C22	−7.63 (19)	F1—C24—C25—C26	−179.68 (14)
C21—N5—C18—C17	169.65 (12)	C23—C24—C25—C26	0.4 (3)
C19—N5—C21—C22	−148.02 (13)	C24—C25—C26—C21	0.4 (2)

Symmetry codes: (i) *x*+1, *y*−1, *z*+1; (ii) −*x*+2, −*y*+1, −*z*+2; (iii) −*x*+1, −*y*+2, −*z*+1; (iv) −*x*+2, −*y*+1, −*z*+1; (v) −*x*, −*y*+2, −*z*+2; (vi) *x*−1, *y*+1, *z*; (vii) *x*−1, *y*, *z*; (viii) *x*+1, *y*, *z*; (ix) *x*+1, *y*−1, *z*; (x) −*x*, −*y*+2, −*z*+1; (xi) −*x*+1, −*y*+1, −*z*+1; (xii) *x*−1, *y*+1, *z*−1.

### Hydrogen-bond geometry (Å, °)

**Table:**

Cg2, Cg4 and Cg5 are the centroids of the N1/N2/C7–C10, C1–C6 and C21–C26 rings, respectively.

**Table:**

*D*—H···*A*	*D*—H	H···*A*	*D*···*A*	*D*—H···*A*
C6—H6···O1	0.93	2.32	2.8742 (19)	117
C11—H11B···O3^iii^	0.97	2.41	3.3306 (18)	159
C17—H17A···O3	0.97	2.38	2.7660 (19)	103
C5—H5···Cg5^iv^	0.93	2.86	3.4941 (18)	127
C13—H13B···Cg4^iii^	0.97	2.92	3.7395 (19)	143
C18—H18A···Cg2^viii^	0.97	2.73	3.5079 (16)	138

Symmetry codes: (iii) −*x*+1, −*y*+2, −*z*+1; (iv) −*x*+2, −*y*+1, −*z*+1; (viii) *x*+1, *y*, *z*.

## References

[bb1] Allen, F. H., Kennard, O., Watson, D. G., Brammer, L., Orpen, A. G. & Taylor, R. (1987). *J. Chem. Soc. Perkin Trans. 2*, pp. S1–19.

[bb2] Altomare, A., Burla, M. C., Camalli, M., Cascarano, G. L., Giacovazzo, C., Guagliardi, A., Moliterni, A. G. G., Polidori, G. & Spagna, R. (1999). *J. Appl. Cryst.* **32**, 115–119.

[bb3] Brogden, R. N. (1986). *Drugs*, **32**, 60–70. 10.2165/00003495-198600324-00006 3552586

[bb4] Cremer, D. & Pople, J. A. (1975). *J. Am. Chem. Soc.* **97**, 1354–1358.

[bb5] Farrugia, L. J. (1997). *J. Appl. Cryst.* **30**, 565.

[bb6] Farrugia, L. J. (1999). *J. Appl. Cryst.* **32**, 837–838.

[bb7] Pople, J. A. & Beveridge, D. L. (1970). *Approximate Molecular Orbital Theory.* New York: McGraw Hill.

[bb8] Sheldrick, G. M. (2008). *Acta Cryst.* A**64**, 112–122. 10.1107/S0108767307043930 18156677

[bb9] Stoe & Cie (2002). *X-AREA* and *X-RED32* Stoe & Cie, Darmstadt, Germany.

[bb10] Şüküroğlu, M., Küpeli, E., Banoğlu, E., Ünlü, S., Yeşilada, E. & Şahin, M. F. (2006). *Arzneim.-Forsch. Drug Res.* **56**, 337–345. 10.1055/s-0031-1296731 16821644

